# Fate of Residual Pesticides in Fruit and Vegetable Waste (FVW) Processing

**DOI:** 10.3390/foods9101468

**Published:** 2020-10-15

**Authors:** Tri Thanh Nguyen, Carmen Rosello, Richard Bélanger, Cristina Ratti

**Affiliations:** 1 Soils and Agri-Food Engineering Dept, Institute of Nutrition and Functional Foods, Université Laval, Quebec City, QC G1V 0A6, Canada; thanh-tri.nguyen.1@ulaval.ca; 2 Chemical Engineering Group, Chemistry Department, Universitat des Iles Balears, Palma, 07122 Mallorca, Spain; carmen.rossello@uib.es; 3 Soils and Agri-Food Engineering Dept, Université Laval, Quebec City, QC G1V 0A6, Canada; 4 Plant Science Dept, Université Laval, Quebec City, QC G1V 0A6, Canada; Richard.Belanger@fsaa.ulaval.ca

**Keywords:** pesticide, fruit wastes, vegetable wastes, drying, extraction, intensification technologies

## Abstract

Plants need to be protected against pests and diseases, so as to assure an adequate production, and therefore to contribute to food security. However, some of the used pesticides are harmful compounds, and thus the right balance between the need to increase food production with the need to ensure the safety of people, food and the environment must be struck. In particular, when dealing with fruit and vegetable wastes, their content in agrochemicals should be monitored, especially in peel and skins, and eventually minimized before or during further processing to separate or concentrate bioactive compounds from it. The general objective of this review is to investigate initial levels of pesticide residues and their potential reduction through further processing for some of the most contaminated fruit and vegetable wastes. Focus will be placed on extraction and drying processes being amid the main processing steps used in the recuperation of bioactive compounds from fruit and vegetable wastes.

## 1. Introduction

Fruits and vegetables are key elements of a healthy human diet, owing to their high proportion of fibers, vitamins and minerals. In 2010, 6.7 million deaths worldwide were attributed to a low intake of fruits and vegetables causing poor health and a higher risk to develop non-communicable illnesses [1]. In agreement with global trends, fruit and vegetable production rose in 2009 to above 500 and 850 MMT, respectively, and their waste due to primary production, to 40 and 70 MMT [2]. Fruit and vegetable waste arise mainly from tricky processing and inadequate handling of the produce. At the same time, a Swedish study [3] determined that, among the diverse types of produce, apples, tomatoes, peppers and grapes are the ones generating almost 50% of the wastage at the supermarket level. From all this wasted biomass, the development of interesting by-products with applications in food, cosmetic and pharmaceutical industries could be a promising pathway to reach a resource-efficient circular economy [4].

Currently, pesticides are commonly employed to ensure successful fruit and vegetable production. However, their often large-spectrum biocide activity and potential risk to the consumer represent a growing source of concern for the general population and environment [5]. Over the past two decades, many of the most toxic pesticides have been withdrawn from agricultural and/or household practices. Yet others, such as organophosphate insecticides, are still applied to certain crops [6].

A great deal of attention nowadays surrounds the so-called ‘dirty dozen’, a list of 12 fruits and vegetables with the highest concentration of pesticides. Strawberry, apple, grape, tomato and potato figure prominently among them [7]. Handling after harvesting may markedly decrease the pesticide residues in most fruits and vegetables for human consumption, as a result of the peeling and washing processes [8]. On the other hand, the non-edible parts of fruits and vegetables after processing constitute around 10 to 60% of the total weight of the product, and are composed of peel, skin, seeds, sheaths, etc. [9]. Skin and peel are the main constituents of these wastes, representing more than 50% [2]. Thus, the content of agrochemicals in waste of fruits and vegetables should be monitored, especially if originating from peel and skin, and eventually minimized before or during further processing, targeting the separation or concentration of bioactive compounds from it. It is important to point out that health problems due to pesticide intake are not only related to the toxicity level of the agrochemicals but also to their concentration and exposure time.

The general objective of this work is to review the initial levels of pesticide residues in fruits and vegetables and their potential reduction/increase through further processing for some of the ‘dirty dozen’ fruit and vegetable wastes. Focus will be placed on extraction and drying processes being among the main processing steps used in the recuperation of bioactive compounds from such wastes.

## 2. Bibliographic Research Methodology

A literature search on pesticide residues in fruit and vegetable wastes (FVW) and the effect of extraction and drying processes on reducing/increasing pesticide residues was carried out on the ScienceDirect, PubChem, and Google Scholar database. The combination of ‘keywords’ used for the search includes ‘pesticide residues’, ‘fruit waste’, ‘vegetable waste’ AND ‘processing’, ‘pesticide residues’ AND ‘extraction’ OR ‘drying, AND ‘pesticide residues’ AND (‘PEF’ OR ‘Ultrasound’ OR ‘Microwaves’). The literature reference sections of the retrieved articles were used to find more studies that might have been missed out during the literature search.

## 3. Pesticides in Fruits and Vegetables

### 3.1. Classification and Properties

“Pesticide” is a term for all insecticides, herbicides, fungicides, rodenticides, wood preservatives, garden chemicals and household disinfectants that may be used to kill some pests. Pesticides may be classified based on several parameters, depending on the needs. In any case, the three more popular pesticide classifications are based on (i) the mode of entry to the plant, (ii) the pesticide function and the pest organism they kill, and (iii) the chemical composition of the pesticide [10].

Based on their chemical composition, pesticides can be classified in the groups of organochlorines, organophosphates, carbamates and pyrethroids. Table 1, Table 2, Table 3 and Table 4 provide information on the previous four groups of pesticides, including health and environmental hazards, chemical formulas and the main fundamental properties of selected common pesticide compounds.

Table 1 shows the most common examples of pesticides within the organochlorine group, which are organic compounds with five or more chlorines atoms attached. They are widely used as insecticides, such as dichlorodiphenyltrichloroethane (DDT), that is effectively used for the control of malaria in many tropical developing countries [21]. However, owing to the nature of their characteristics (volatile, low polarity, low aqueous solubility, and high lipid solubility), these pesticides have a long-term residual permanence in the environment after application. Moreover, their bioaccumulation and toxicity characteristics may cause hypertension, cardiovascular disorders and other health-related problems in humans, resulting in their ban in many developed countries [22].

Another group of pesticides, the organophosphates (Table 2), includes organic compounds that contain phosphodiester bond in their basic structure. As a result, they easily decompose when applied on plants, and soil, causing reduced environmental pollution. Their activity is mainly directed toward the inhibition of acetylcholinesterase, which controls the functions of the nervous system [23]. The most common examples of organophosphate pesticides shown in Table 2, have higher water solubility than those in the organochlorines group, but they are also more soluble in organic solvents.

Most pesticides belonging to the carbamate group (Table 3) are highly soluble in common organic solvents. Their activity is similar to those of organophosphate pesticides, as they also inhibit the enzyme acetylcholinesterase [23]. Pyrethrin and pyrethroid (Table 4) have low water solubility, while others such as deltamethrin are not water soluble. Nevertheless, they easily decompose when exposed to light, and are only slightly toxic to mammals and birds, so they are generally considered as the safest insecticides for use in food consumption [10].

Pesticide physicochemical properties shown in Table 1, Table 2, Table 3 and Table 4, such as solubility and vapor pressure, lead to differences in pesticides plant uptake, environmental distribution, as well as their elimination during fruit and vegetable harvesting and processing. For example, solubility of pesticides in water or organic solvents, plays a key role on their ability to be dissolved in solvents with different polarities during extraction of valuable compounds [13]. Moreover, pesticides with higher vapor pressure are more likely to volatilize i.e., during drying as water evaporates, while low vapor pressure pesticides tend to accumulate in liquid phases, soil or biota. In a study by Sood et al. (2004) [24], the percentage of dimethoate pesticide residue left after the drying step during green tea manufacture was the lowest (23.4%) related to its higher vapor pressure. For pesticides with low water solubility, high vapor pressure contributes markedly in decreasing their content, such as 19% loss of tridemorph residue during black tea drying, which was higher than hexaconazole, propiconazole, and carbendazim residues, less than 7% [25].

### 3.2. Toxicity and Maximal Allowed Concentration

Pesticides are reported to have an impact on human health and have been linked to illnesses, ranging from acute ailments to chronic diseases, such as cancer, reproductive disorders, and endocrine-system dysfunctions [26]. Table 1, Table 2, Table 3 and Table 4 summarize some health and environment issues for some selected pesticides. For these reasons, it is widely agreed that the use of pesticides should be carefully monitored to prevent negative effects on health, ground water sources and the environment.

Maximum residue limits (MRLs) (expressed in µg kg^−1^) are the highest levels of residues expected to be found in food products when the pesticide is used in accordance with its label [27]. The MRLs are systematically set far below levels considered to be unsafe for humans, meaning that food residues containing higher levels than the MRL are not necessarily unsafe for consumption [28].

MRLs were established and recommended by the World Health Organization (WHO) and the Food and Agriculture Organization (FAO). MRLs are also subject to specific legal requirements in most countries, such as those set by the Pest Management Regulatory Agency (PMRA) in Canada, the Food and Drug Administration (FDA) in the United States, and the European Commission (EC) in Europe.

Table 5 shows a comparison of MRLs (pesticides mentioned in Table 1, Table 2, Table 3 and Table 4 for fruits and vegetables popular in Canada, i.e., apples, potatoes, tomatoes and strawberries), established by the PMRA, FDA, and EC. Generally, MRLs set by the EC are lower than in North America, where a lower tolerance for pesticide residue limits in fruits and vegetables is applied, i.e., MRLs of cypermethrin on strawberries was set by Canada to be twice as high as the EC, while in the US, MRLs of carbaryl on apples are 1200 times higher than those in Europe. As well, MRLs for malathion and diazinon are 25 to 400 times higher in North America than in Europe. However, in other cases, MRLs from the US and EC are comparable, such as those for DDT and glyphosate in tomato, or permethrin in potato. In rarer instances, lower MRLs are imposed in the US than in Europe, such as in the case of glyphosate in potato and strawberry, cypermethrin in tomato, and deltamethrin in potato.

However, sometimes pesticides that are banned in Canada or the US, are still permitted in Europe. For example, in the case of organochlorines, lindane was banned in Canada from December 2004 [29] because of its toxicity and persistence in the food chain, together with aldrin, dieldrin, and chlordane, while the EC still allows them, albeit in very low MRLs, sometimes three to ten times lower than the accepted values by the FDA, depending on the type of fruits and vegetables. For organophosphates, parathion and methyl parathion are forbidden in Canada and not applied in United States on fruits and vegetables mentioned in Table 5, because of increasing concerns regarding hazards to wildlife and human health, while still being accepted in Europe. Aminocarb in the carbamates group is not allowed in the listed fruits and vegetables of Canada, US and Europe, because of its toxicity for human health and environment. For carbaryl residues, MRLs set by the EC are very low, from 20 to 1200 times lower than US and Canada. Similar situations are described in Table 5 for pyrethrins and members of the pyrethroid group.

### 3.3. Fruits and Vegetables with the Highest Presence of Pesticides

The list of twelve fruits and vegetables with the highest amounts of pesticide residues (named “dirty dozen”) is annually published by the Environmental Working Group (EWG), a nonprofit organization. In 2019, the “dirty dozen” ranking was composed, in order of importance by: strawberry, spinach, kale, nectarine, apple, grape, peach, cherry, pear, tomato, celery, and potato. These products were found to have higher levels of pesticides than all other ones over the year [7].

From the data obtained by the United Stated Department of Agriculture (USDA) for their Pesticide Data Program in recent years, strawberry may contain as many as 45 different types of residues. Other fruits and vegetables also present a high number of pesticide residues, such as apples (47), grapes (56), cherries (42), tomatoes (35), potatoes (35), sweet bell peppers (53), etc. Among them, tetrahydrophthalimide (THPI), a metabolite from the non-systemic fungicide-captan, was found in 55% of strawberry samples, while permethrin, an insecticide of the pyrethroid family, dominated in 52% of spinach samples; formetanate hydrochloride, a carbamate pesticide that inhibit cholinesterase, in 53% of nectarines; diphenylamine (DPA), an aromatic amine used as a scald inhibitor for apples, was found in 83% of samples; imidacloprid, a systemic insecticide, in 48% of grapes; fludioxonil, a non-systemic fungicide, in 48% of peaches; boscalid, a non-systemic fungicide, in 65% of cherries; pyrimethanil, an anilinopyrimidine class of fungicides, in 40% of pears; endosulfan, an organochlorine insecticide and acaricide, in 17% of tomatoes; and chlorpropham, a carbamate herbicide, in 80% of potato samples [30].

### 3.4. Pesticide Application and Physical Location in Fruits and Vegetables

Pesticides are mainly sprayed on fruits and vegetables and accumulate often on the outer peel or skin, the cuticle [31]. The pesticide could be adsorbed by the plant surface (waxy cuticle and root surfaces) and enter the plant transport system (systemic) to protect it from pests that penetrate the skin; other pesticides may stay on the surface of the plant (contact). While still on the surface of the crop, the pesticide is exposed to environmental factors such as wind and sun, and may be washed off during rainfall. As well, they can undergo volatilization, photolysis, or chemical and microbial degradation [32].

As mentioned above, pesticide residues commonly accumulate on the peel or skin. For instance, thiabendazole and ortho-phenyl-phenol was detected in harvested citrus fruit peels [33], residues of organochlorine pesticides (DDT and its derivatives, lindane, HCB) and organophosphorous pesticides (pirimiphos-methyl, dimethoate, malathion) were detected in potato skins [34]. Difenoconazole was found to be present in tomato skin [35]. In another study, Abou-Arab (1999) [36] reported that hexachlorobenzene, o.p-DDD, p.p-DDD, dimethoate and profenofos were present in the skin of tested tomato, where they were two to seven times higher than in their pulp. In the same study, organophosphate (dimethoate and profenofos) residues were also reported in the seeds of tomato. In the same manner, hexythiazox, a non-systemic acaricide, is applied on the surface of fruits by contact mode, and although it can be easily washed off, it is also absorbed in the pulp of treated strawberry [37].

### 3.5. Pesticide Analytical Determination

As mentioned previously, pesticides applied in fruits and vegetables are classified based on various criteria, such as mode of entry, mode of action or chemical composition and characteristics [10]. Accordingly, its residues contain not only their main compounds, but also their metabolites and/or degradation products, which have different physicochemical characteristics (vapor pressure, polarity, solubility). This multicompound presence results in difficult and complex methods to isolate pesticide residues in micro-quantities from fruit and vegetable matrices.

Pesticide residues in fruits and vegetables are analyzed through two steps: (a) extraction and clean-up of the target analytes from the matrix, and (b) determination of the target analytes [38]. For the first step, various techniques could be used, such as liquid-liquid extraction (LLE), solid phase extraction (SPE), solid phase micro extraction (SPME), and QuEChERS (quick, easy, cheap, effective, rugged, and safe) extraction.

Liquid-liquid extraction (LLE), also known as partitioning, is a separation process consisting of the transfer of a solute from one solvent to another, the two solvents being immiscible or partially miscible with each other [39]. Organic solvents such as acetonitrile, ethyl acetate, chloroform, hexane, 1,2-dichloromethane, etc. are usually used in LLE methods for the determination of pesticide residues in food and the environment, due to their good solubility in several immiscible liquids, such as in water and organic solvents. For instance, de Pinho et al. (2010) [40] used a mixture of acetonitrile and ethyl acetate (6.5 mL:1.5 mL) as the solvent for extraction of chlorpyrifos, λ-cyhalothrin, cypermethrin and deltamethrin in honey samples. Acetonitrile was also used as an extraction liquid for carbamates (aldicarb, carbofuran and carbaryl) in water samples [41].

Solid-phase extraction (SPE) is one of the most widely used packing column or cartridge extraction methods. Analytes are initially adsorbed onto suitable solids depending on their interaction. Then, a selective organic solvent is used to remove interferences, and then another solvent is selected to elute out the target analytes. Advantages of SPE methods are the reduction of solvents quantities, short concentration time, and improved yield recovery [42]. A study by Torreti et al. (1992) [43] analyzed 15 organochlorine pesticide residues from samples of animal feed, using a C18 SPE column as clean up procedure providing high recovery (70–100%). In another study, a multiresidues method for analysis of 90 pesticide residues with different physicochemical properties in fruits and vegetables was developed, where a polystyrene divinylbenzene column (LiChrolut EN) was used as an effective SPE method for clean-up and pre-concentration procedures of the pesticides from water-diluted acetone extracts [44]. In a recent study, a combination of graphitized carbon black and primary secondary amine (GCB/PSA) was used as SPE method for clean-up process, followed by the injection of fruit and vegetable extracted samples into the UHPLC-TOF/MS to analyze 60 targeted pesticides [45].

Solid phase microextraction (SPME) is a simple, low cost, easily automated and on-site sampling method when compared to SPE. It involves two processes: analytes are separated from the sample by the coating, and the desorption of concentrated analytes are analyzed by an analytical instrument [46]. Because of its advantages, particularly that of being solvent-free, SPME formed by a silica fiber coated with a polyacrylate (PA) film was used in clean-up procedures, followed by GC-MS, for the determination of organophosphate pesticides in wine and fruit juices [47], and of 14 pesticide residues (clofentezine, carbofuran, diazinon, methyl parathion, malathion, fenthion, thiabendazole, imazalil, bifenthrin, permethrin, prochloraz, pyraclostrobin, difenoconazole and azoxystrobin) in mango fruit [48].

The QuEChERS (Quick, easy, cheap, effective, rugged, and safe) sample preparation is a simple, fast, and inexpensive method, originally described by Anastassiades et al. (2003) [49], for the determination of pesticide residues in fruits and vegetables. The QuEChERS technique involves two steps: a liquid-liquid extraction and dispersive solid-phase extraction clean-up. The samples pre-treated using QuEChERS are clean enough to be analyzed using gas or liquid chromatography [50]. Due to the numerous advantages of this method, it was used by many researchers. In a recent study, QuEChERS process provided satisfactory results with high recovery (acceptable ranges) of 72 pesticides in carrot, corn, melon, rice, soy, silage, tobacco, cassava, lettuce and wheat [51], and 11 fungicides, three insecticides in strawberry by-products [52]. In another research on optimization of the clean-up step of QuEChERS method in coffee leaf extracts [53], it was possible to analyze 52 pesticides by LC-MS/MS. For this, the clean-up procedure of QuEChERS method was modified with different combinations of adsorbents, resulting in high recovery (>70%). Recently, the combination of modified QuEChERS method by adding of acetonitrile with 0.1% formic acid, followed by UHPLC-MS/MS determination, was applied by Lee et al. (2018) [54] for a multiresidue analysis of 310 pesticides in brown rice, orange, and spinach, which resulted in 87–89% of the pesticides at spiking level of 10 ng g^−1^ met the acceptability criteria of DG-SANTE guidelines (recovery 70–120%, and RSD ≤ 20%).

For the second step in pesticide analytical determination, i.e., the detection or analysis of target analytes (pesticides) in foods, numerous conventional analytical methods are used such as gas chromatography (GC), high performance liquid chromatography (HPLC), or more delicate including gas chromatography associated with mass spectrometry (GC-MS), liquid chromatography associated with mass spectrometry (LC-MS), and ultra-high performance liquid chromatography tandem mass spectrometry (UHPLC-MS/MS).

For volatile pesticides, which can be easily vaporized, GC is a popular separation method applied in several studies. It is usually coupled with specific detectors, including flame ionization detector (FID), as for the analysis of organophosphorus pesticides in onion, grape and apple juices [55], or pyrethroid pesticides in vegetable oils [56]. Electron capture detector (ECD) has been used as well in the determination of chlorpyrifos-methyl, fenitrothion, procymidone and vinclozolin on peach [57], and the flame photometric detector (FPD), to determine 11 organophosphorus pesticide residues on cabbage, kale and mustard samples [58]. Mass spectrometer detectors (MS and tandem MS) are also popular choices for pesticide determination, as MacLoughlin et al. (2018) [59] analyzed 35 commonly used pesticides by GC-MS, and 381 different types of pesticides in grapes were monitored by GC/MS-MS [60].

On the other hand, for high polarity and non-volatile extracted analytes, HPLC analytical techniques are preferably used as an effective separation method. It can be coupled with detectors such as UV in the case of analysis of pyrethroid residues in fruit and vegetable samples [61] or MS and tandem MS in the determination of malathion, diazinon, imidacloprid and triadimefon in fruit juices (apple, cherry, raspberry, orange and pineapple) [62].

In recent years, UHPLC started the use of smaller stationary-phase particle size (≤2 µm) than those used in classical LC (3–5 µm), for the detection of 21 pesticide residues in tomato and sweet pepper samples, coupled with tandem mass spectrometry (UHPLC-MS/MS) [63]. In another study, time-of-flight mass spectrometry (TOF-MS) was combined with UHPLC to detect 60 pesticides in 286 vegetable and fruit samples [45].

## 4. Fruit and Vegetable Waste (FVW)

### 4.1. Common Types of Fruit and Vegetable Wastes

Fruits and vegetables generate at least 10 to 60% of waste materials that are composed of leaves, roots, tubers, skin, pulp, seeds, peel, pomace, etc. [9]. Their percentage and type of waste vary from process to process, and fruit and vegetable types, such as sliced apples, generated 11% of seed and pulp as waste; papaya yielded about 9% of peel, 7% of seeds, 32% unusable pulp as wastage products, and mangos produced 14% of seeds, 11% of peels, 18% unusable pulp [2]; while in the case of apple juice processing, apple pomace as a discarded solid residue dominated 25–30% of the total processed fruits [64]; grape pomace, a wastage of wine production, mainly containing of seeds and peels, contained about 20% of the total weight [65]. Potato peel, a wastage of potato processing, can vary from 15 to 40%, depending on the procedure applied to remove the skin, i.e., steam, abrasion or lye peeling, while in tomato juice processing, only 3–7% of the raw material is lost as waste [66].

### 4.2. Potential Applications of Fruit and Vegetable Wastes

Since fruit and vegetable wastes are a source of dietary fiber and bioactive compounds such as phenolics or vitamins, their utilization received an increased attention recently for the application as functional ingredients in the food industry [67], or in other industrial applications such as pharmaceutical and/or nutraceuticals, healthcare and chemicals [2].

For instance, by-products extracted from potato peel waste [68], apple peel [69] and passion fruit peel [70] have high antioxidant activity. They can also be used as a base for fermentation reactions, and in healthy and functional food production as a dietary fiber source. Furthermore, in another study, the viscosity and emulsifying properties of pectin extracted from potato peel waste could be improved when treated with high pressure processing [71].

Apple pomace, a by-product of apple processing, has the potential to be incorporated as a natural stabilizer and texturizer in yogurt fermentation by increasing the gelation pH, shortening the fermentation time, and developing a firmer and more consistent yogurt gel during cold storage [72]. Moreover, apple pomace was incorporated into wheat flour as a fiber source to improve the rheological characteristics of cake batter [73]. The characterization of polyphenols, proantocyanidin profiles and antioxidant activity of pomace from different varieties of grapes [74,75] pointed out the possible exploitation of winemaking by-products as inexpensive and easily available sources of bioactive compounds. Different classes of polyphenols and ursolic acid were extracted from grape pomace as well [76], which was also used to enrich yogurt and salad dressing by increasing dietary fiber, total phenolic content, and delaying the lipid oxidation of samples during refrigeration storage [77]. In addition, grape seed flour can be used as an ingredient for cereal bars, pancakes and noodles products [78]. The availability and the potential of wine by-products, grape pomace and stems, obtained from ten different grape (*Vitis vinifera* L.) varieties as raw materials for the production of dietary fiber concentrates, were in addition evaluated with regard to their potential incorporation as dietary fiber concentrates into the food chain [79]. Minjares-Fuentes et al. [80,81] reported that an ultrasound-assisted procedure for the extraction of pectins from grape pomace could be a good option for the extraction of functional pectins and hemicellulosic polysaccharides from grape pomace at the industrial level.

Umaña et al. (2020) [82] investigated the revalorization of mushroom by-product (stalks of A. bisporus) by extracting its components (ergosterol and antioxidant components from mushroom by-products and the attainment of a β-glucan rich residue).

Zhang et al. (2018) [83] studied the effects of dynamic high-pressure micro-fluidization (DHPM) on the physicochemical properties and rheological properties of pectin extracted from black-cherry tomato waste (pomace). In other studies, tomato peel was reported to have amino acids and fatty acids, besides a high content of antioxidants such as flavonoids, phenolic acids, lycopene, ascorbic acid and minerals (Ca, Cu, Mn, Zn, and Se) [84,85,86].

Sójka et al. (2013) [87] reported that a by-product of strawberry juice production (strawberry press cake), consisting of 40% seeds, 4% sand, and about 55% exhausted strawberry flesh, is an important source of nutrient and polyphenolic composition, including proanthocyanidins, ellagitannins, and especially dimeric agrimoniin. Furthermore, the ingestion of extracts of industrial strawberry pomace showed beneficial health properties with positive changes in the population of intestinal microflora [88].

Moreover, pectin was also extracted from several fruit and vegetable waste sources, such as orange peel [89], melon peel [90], banana peel [91,92], peach pomace [93], potato peel [71], and berry fruit residues [94]. Pectin from berry residues were found to have high quality and purity parameters that make it suitable for commercialization, either as an additive in food or for the elaboration of medicine-related compounds [94].

In addition to valuable bio-functionalities, fruit and vegetable wastes also have increased traditional nutritional values [84,85,86]. For example, the waste of seven types of underground vegetables (beet, turnip, carrot, sweet potato, radish, potato, ginger) was found to contain vitamin C levels ranging from 44 to 123 mg/100 g, riboflavin from 0.3 to 0.8 mg/100 g, thiamin around 0.4 mg/100 g, and niacin from 0.2 to 1.6 mg/100 g, and a high content of calcium, sodium, magnesium, iron, manganese, zinc, potassium and phosphorus [95].

### 4.3. FVW by-Products Processing

Among the multiple potential applications shown for FVW, an increased special attention is recently being paid to the extraction of polyphenols and antioxidants from agri-food by-products and converting these extracts into stable powders.

Grape pomace or wine marc is one such agri-food processing waste, which is generated in the range of 5–9 million tons per year worldwide by the wine industry. A recent manuscript deals with possible uses for red wine processing waste, proposed to convert grape pomace into powder with a processing line, during which pomace was separated to be air-dried in an oven at temperatures 45–50 °C for 72 h to remove the remaining mixture of water and alcohol. Then, dried grape pomace was ground after removing seeds and stems and was used to mix with refined wheat (5 to 20%) in cookies to increase their polyphenol content [96]. Another application of grape by-products is the obtention of dietary and phenolic concentrates. For this, the freeze-dried pomace was ground into a homogeneous powder. Acetone (50% aqueous) was used as a solvent for phenolic and dietary fiber extraction. Acetone was evaporated at 60 °C under vacuum. Phenolic compounds were purified with butanone by solvent fractionation process and then freeze-dried again, while dietary fiber concentrate was treated by freeze-drying of the extracted solid residue [97]. In another study, the antioxidant extraction process from grape stalk, in order to quantify the influence of the previous drying operation on extraction kinetics, was evaluated by Garcia-Perez, et al. (2010) [98].

For the extraction/concentration of polyphenols from apple waste, the dried pomace was milled and sieved through a 20-mesh (0.84 mm) sieve into a homogeneous powder. Five (5) g of dried powder was mixed with 200 mL of 70% alcohol (solvent for polyphenol extraction). Alcohol was evaporated under reduced pressure, and then polyphenol compounds were concentrated in the powder by freeze-drying [99]. In another study, apple pomace was used to obtain pectin by extraction [100]. For this, nitric acid solution (pH = 2.5) was employed as an effective solvent for extraction at 80 °C during 1 h, and ethanol was used as agent in pectin precipitation, then pectin was filtered and vacuum dried at 45 °C to constant weight, and finally ground.

Furthermore, potato peel waste was also analyzed to be converted into an antioxidant powder. The phenolic compounds, antioxidant and antiviral activities of extracts were evaluated in dried potato peel (dried at 45 °C for 24 h and then powdered). Absolute ethanol with 5% acetic acid (95:5 ratio) was used as an extraction solvent for 72 h, and then the samples were filtered and concentrated by freeze-drying [101].

As shown in the few previous examples of processing lines for extracting valuable compounds from FVW, but also in many other cases found in the literature [102], extraction and drying processes are crucial operations which are always present in the further processing of plant-based food wastes.

## 5. Drying and Extraction in FVW by-Products Processing

Drying and extraction processes play an important role in the elimination and separation of contaminants and moisture content, by concentrating bioactive compounds from waste. They also prevent undesirable biochemical changes during storage for further processing. Salim et al. (2017) [102] reviewed conventional and emerging technologies for the conversion of FVW into value added products, where diverse drying and extraction methodologies were thoroughly described and analyzed. Moreover, other techniques, such as enzyme-assisted, subcritical water, microwave-assisted [103], and ultrasound-assisted extraction were proposed by Adetunji et al. (2017) [104], to improve the efficiency of industrial extractions from FVW. In the following paragraphs, a brief description of the principles underlying drying and extraction processes, and intensification methodologies that sometimes assist these processes, will be presented, in order to better understand how these systems could impact in the retention/disposal of pesticide residues.

Drying is a widespread technique in the food industry and a subject of continuous interest in food research. Most food products are dried for improved milling or mixing characteristics in further processing [105]. However, negative changes in food quality may occur during air-drying [106,107]. During the drying of food products, and especially of by-products such FVW, the moisture to be removed does not consist of only one component [108], but of a mixture of two or more components (multicomponent mixture), which could be thermodynamically non-ideal liquid solutions. The material to dry may contain ethanol, acetone, acids such as acetic or nitric, and even several different pesticides, competing with water in the vaporization process. The interaction between these compounds in the mixture through hydrogen bonding, dipolar interaction, or electrostatic interaction, and their different physicochemical characteristics (vapor pressure, boiling point, dissolution in water), will differentiate normal drying (where just water is evaporating) with multicompound drying, where the components can be evaporated, degraded, or co-evaporated together with water. This inter-relationship of compounds in the mixture affects multiple phenomena, such as the sorption behavior, gas-solid mass transfer, and the multicomponent vapor-liquid equilibrium. Furthermore, during the process, the wet bulb temperature changes, the initial composition of moisture, the identity and initial composition of the liquid system, the vapor pressure of different compounds, and the characteristics of the solid can influence the evaporation of selected components [109]. In the multicomponent drying case, drying behavior and its kinetics could not be simply explained by Fick’s law [108] and, therefore, other mathematical representations of the phenomenon, such as the Maxwell-Stefan equations, should be used [110]. The diffusion behavior of solvents in a mixture during evaporation differed from when they are alone; it may cause a decrease or increase in the drying time, depending on their solubility, diffusion coefficient, which could be influenced by their concentration and partial pressure [111], or their ratio of gas-side mass transfer coefficient between them in mixture, affecting which component is removed preferentially [112]. The interaction between moisture and solid also affects the drying behavior (drying rate or diffusional paths, composition curves) when other solvent, such as, for instance, isopropyl alcohol is combined with water during drying [113]. Ho and Udell (1995) [114] investigated the influence of different binary mixture systems (toluene with o-xylene, benzene with o-xylene, and toluene with octane) at variable concentration and applied time on reducing of hydrocarbon contaminated soil. In pharmaceutical applications, during the drying of itraconazole, drying kinetics and dried particle morphology were affected by various weight fractions of binary solvent mixture of dichloromethane and ethanol [115]. Moreover, the pervaporation of a multi-compound (binary, ternary) mixture solvent from a membrane or solid state, as described by Heintz and Stephan (1994) [116], could also be explained by the mutual interaction effect between components (water and organic compounds); this frictional interaction force may lead to decrease or increase of component flux in diffusion. The friction coefficient is influenced by the size and shape of molecule of component during its movement through membrane, and this coefficient is accounted for, in part, in the modified Maxwell-Stefan equations [111].

Extraction is an important separation process used in various food processing applications as well. In this process, a desired component in a solid/liquid phase is separated by contact with a suitable solvent. Thus, the compositions of both phases change simultaneously during extraction, until equilibrium is reached. These phases are subsequently separated, and the desired component is recovered from the liquid phase [107]. Extraction constitutes a main processing stage to produce certain food products (oils, sugars), or to isolate desired compounds (antioxidants, vitamins). It could be useful to remove contaminants and other undesirable components and toxins present in food sources. Commonly used solvents in the extraction of food components are water, ethanol (or ethanol-water mixtures), hexane, and carbon dioxide, but the trend is toward the use of natural chemicals [117]. The rate of extraction is influenced by the solid-liquid interface area, the concentration gradient (to ensure a complete extraction, a sufficient gradient must be maintained between the concentration of solute at the surface of the solid and in the solvent), and by the mass transfer coefficient (an increase in temperature increases the rate of solution of the solute in the solvent and also the rate of diffusion of solute through the solution) [107,118]. Concentration changes as a function of extraction time could be represented by empirical-type models, such as Peleg’s model [119], Page’s or Weibull’s model [82,120,121,122]. Additionally, in liquid-solid extraction, the solubility of compounds in different solvents, and the polarity characteristic of solvents are factors that influence the yield of extracted compounds [123,124].

### 5.1. Process Intensification (PI)

Different strategies are necessary to transform waste into valuable by-products. As mentioned before, intense work has been carried out focusing on the selection, characterization and stabilization of different agro-food by-products [125,126]. In this sense, drying and extraction are key processes for such valorization, but they could pose various techno-economic and environmental challenges, including low product yields, excessive energy consumption, or valuable compound deterioration such as carotenoids or polyphenols. As well, recent trends in extraction techniques have largely focused on finding solutions that minimize the use of solvents, sometimes causing health and environmental threats. Many of those challenges can be addressed by the application of innovative intensification technologies, such as pulsed electric field [127], ultrasound [128], or microwave [103], among others. The aim of these techniques is the improvement of traditional processes by increasing production yields, reductions in equipment size, energy use and waste, and increasing product quality. In terms of process safety, the reduction of plant size results in a smaller volume of toxic and flammable inventories within processes, thereby reducing the possibility of major explosions [129]. However, if intensification is to be applied to food production in the coming years, an integral analysis of the application of these new technologies need to be explored in detail, so as to offer sustainability in processing and cost-effective production of high-quality extracts.

Over the years, the implementation of PI has evolved into two distinct classifications involving the application of intensification technologies as pretreatments prior to processing, or else, during the process itself. In the following paragraphs, descriptions of some of the most used PI technologies applied to drying and extraction processes and their food applications will be individually presented.

#### 5.1.1. Pulsed Electric Field (PEF)

Pulsed electric field (PEF) is an emerging technology with a wide variety of applications in the food and biotechnology sectors. It has been originally applied as a non-thermal process to the inactivation of bacteria, molds, and yeasts with promising results; other applications include the inactivation and modification of enzymes with negligible or minimum changes to the sensory, physicochemical, and nutritional characteristics of the product [107]. Despite the fact that PEF has been initially used in food processing as a separate and independent process, it can also be utilized as a pretreatment method, in order to enhance the subsequent process kinetics or to modify a quality of final products [130].

In a PEF system, the energy derived from a high voltage power supply is stored in an energy storage capacitor bank and discharged through a food material in a treatment chamber by the supporting of pulse generator to generate the necessary electric field in the food [131]. Important parameters that determine PEF processing impact are the treatment time and the electric field intensity (kV cm^−1^), which is the ratio between the peak voltage (kV) and the gap distance (cm) between the electrodes in the treatment chamber.

PEF causes an irreversible loss of the membrane function as a semipermeable barrier between the bacterial cell and its environment [132]. Moreover, PEF treatment also leads to a cell membrane disintegration or electroporation phenomenon, intensifying any process based on mass transfer in cellular systems [133], and increasing the vibration and rotation of polar molecules [134].

Regarding drying, interesting technologies related to the application of moderate-continuously (MEF) or high voltage-pulsed (PEF) electric fields were investigated as a pre-treatment of different drying processes, such as osmotic dehydration, vacuum drying convective drying or freeze-drying, significantly increasing the dehydration kinetics [135,136,137]. These treatments can induce the formation of pores (permanent or reversible) in the cell membrane, facilitating the mass transport, such as in the research by Ostermeier et al. (2018) [138], prior to the convective drying of onion. Cell disintegration and enhanced mass transfer resulted in a 30% reduction in drying time. The effective diffusion coefficient increased from 3.7 × 10^−9^ to 1.8 × 10^−8^ m^2^/s by increasing field intensity up to 1.07 kV cm^−1^. Generally, the greater effect of PEF on the drying rate could be observed when drying was carried out at moderate temperatures. In the case of thermal sensitive foods, the enhancing of the convective drying rate only at moderate temperatures is very important for the keeping both of products’ quality and energy economy, and PEF treatment showed advantages by an increase of the effective moisture diffusivity, allowing a decrease in the drying temperature from 70 °C to 50 °C during convective drying of potato, at moderate electric field strengths (E = 300–400 V cm^−1^) [139]. However, the electroporation effect on cell membranes by the PEF treatment results in cell membrane breakdown, and natural compounds such as polyphenols, β-carotene and others may be released and lost by oxidation, or a color change by browning reactions during drying may occur. For solving this problem, oxidation reducing agents such as sodium sulfite can be added to protect natural compounds from oxidation [140]. Regarding PEF pre-treatment influence on antioxidant capacity, no significant changes have been reported for the convective drying of blueberries [141].

PEF has also been applied as a pre-treatment for extraction yield improvement through the electroporation phenomenon, for instance in the case of juice extraction from whole fruits, where the yield increased by 25% for orange, 37% for grapefruit and 59% for lemon [142]. Moreover, PEF treatment improved the polyphenol content after extraction [143], where the yield of total polyphenols extracted from orange peel increased from 20% to 159% for PEF pre-treated at 1 to 7 kV/cm, respectively. The quantity of flavonoids (naringin and hesperin) also increased from 1 to 3.1 mg/100 g and from 1.3 to 4.6 mg/100 g of fresh weight orange peel, without and with PEF pre-treatment, respectively. Conditions of PEF treatment played an important role in the change of cellular disintegration index (*Zp*), which was used to determine the effect of PEF conditions to permeabilize samples. In the case of lemon peels, *Zp* values increased to a highest value of 0.55, when the electric field strength and treatment time increased (up to 9 kV/cm, 30 pulses of 3 µs) [144].

Thus, PEF application yields to high-quality and less processed products, but it has a high initial cost for setting up the system. In addition, the requirement of major costs for power supply, the need for a high-speed electrical switch of the pulse generator when operated at a high pulse frequency and large-scale applications, are the main disadvantages of PEF technology [145].

#### 5.1.2. Ultrasound (US)

Ultrasound waves are above the audible range (>20 kHz), with low-intensity ultrasound having frequencies higher than 100 kHz at intensities below 1 W cm^−2^ and high-intensity ultrasound, between 20 and 100 kHz at intensities higher than 1 W cm^−2^ [146]. Low-intensity ultrasound is used to transmit energy through a medium, without or with minimal physical and chemical changes in the material, therefore it can be employed for food analysis and quality control. In contrast, high-intensity ultrasound employs higher power levels for desired physical and chemical properties changes for various bioprocessing applications [147]. Ultrasound waves can be applied both as a pretreatment to the vegetable matrix prior to processing or assisting the actual process, in order to accelerate mass transfer by different mechanisms [148].

In general, applied ultrasound produces alternating compressions and decompressions which affect liquid and solid materials differently. In liquids, the provoked effects are pressure variations and stirring or cavitation. In solid materials, the “sponge effect” is predominant, which produces the release of liquid from the inner part of the particle to the surface and an entry of fluid from outside. Therefore, the forces involved in this mechanical effect could be higher than the surface tension of the water molecules inside the solid, making the mass exchange easier. Moreover, other effects could be occurring, such as changing of viscosity, surface tension, or deformation of solid material [149,150,151].

In terms of the application of US to drying, high intensity ultrasound produces a series of effects that can enhance heat and mass transfer. In fact, US has been applied to intensify convection drying or atmospheric freeze-drying of different products and by-products [128,152,153], achieving important reductions in operation time and energy consumption [154]. In most cases, ultrasound-assisted processing was used as a method to improve appearance characteristics (color, tastes) of dried fruit products by modifying drying kinetics [133], or to preserve bioactive compounds such as polyphenols, anthocyanins and flavonoids [128]. The effect ultrasound on diffusion and mass transport processes during drying can be quantified by the increase in effective diffusivity values, which can be influenced by sample tissue characteristics such as porosity (*ε*) and hardness (H). As a result, when 31 kW m^−3^ of ultrasound power was applied to samples being dried at 40 °C and 1 m s^−1^, a *De* increase of 87% in apple (*ε* = 0.233, H = 25.92N) was obtained, while only 57% was observed in the case of cassava (*ε* = 0.029, H = 38.28N) [155].

As ultrasound-assisted drying reduces the drying time, bioactive compounds content and antioxidant activity should be protected from thermal exposure and better maintained during drying. According to Vallespir et al. (2019) [128], losses of total polyphenol, ascorbic acid, and vitamin E contents in kiwifruit dried with ultrasound (20.5 kW m^−3^) drying at 15 °C were lower than in dried samples without ultrasound application. In another study, when apple was dried by convective drying at temperature 30 °C with ultrasound (18.5 and 30.8 kW m^−3^), the loss of the total polyphenol was lower (34%) than without ultrasound (39%). However, at higher temperatures (50 and 70 °C), ultrasound assistance promoted a higher degradation of polyphenols; 39% loss compared to 20–27% without treatment [156]. Later, the same researchers found out that US can be effectively used in shortening the drying of apples at temperatures below 10 °C without compromising the quality [157]. In general terms, the application of ultrasound can reduce the drying time and protect bioactive compounds only when applied at low temperatures. However, these compounds can be negatively affected when ultrasound-assisted drying is applied at higher temperatures as a result of an increased temperature and thermal exposure in the sample from ultrasound energy [148]. The reader is encouraged to obtain more detailed information from the interesting review article on food drying enhancement by ultrasound [158,159].

Llavata et al. (2020) [160] have compared the influence of different pre-treatments (ultrasound, pulsed electric fields, high pressure processing or ethanol) on the drying process. For this purpose, researchers reviewed the current findings in some of these alternative pre-treatments, addressing their effectiveness on drying enhancement as well as of their impact on quality parameters, such as the retention of bioactive compounds, the color or the texture of the final product.

Regarding separation and recovery of different biocompounds, acoustically assisted solid-liquid extraction [161] has demonstrated high efficiency, by not only improving the recovery yields, but also accelerating the overall process [162]. It has been applied to improve the extraction of compounds with bioactivity [163] and the separation of trace elements [164]. It could also be an alternative to enhancing the sugar release using milder conditions (temperature, type of acid or acid concentration) during the pretreatment of lignocellulose in the second-generation ethanol production [165]. In a study by Caldas et al. (2018) [166], the yield of phenolic compounds in grape skin by ultrasound assisted extraction was twice as high as that obtained by mechanical agitation extraction (80 mg GAE g^−1^), while extraction time was reduced three times. The results from another study also showed that the maximum yield of phenolic compounds by the ultrasound-assisted extraction of grape pomace was achieved within 10 min, compared with 20 h of the industrial batch extraction [167]. The ultrasonic degradation of cell tissue is rapid and occurs within the first minute of treatment, therefore, this intensification technology is usually used for air and light sensitive bioactive compound extraction, such as lycopene from tomato waste [168]. Most recently, Umaña et al. (2020) found that ultrasound-assisted extraction from mushroom by-products yielded up to 2 times higher in ergosterol and 46% in phenolic compounds, depending on ethanol concentration and US power density [82]. Furthermore, in a new comprehensive review of ultrasound assisted extraction, Dzah et al. (2020) concluded that ultrasound assistance is considered nowadays a preferred extraction method, due to its versatility and the ability to use less or no organic solvent, although successful results depend largely on the type of plant material, solvent and the micro-environmental extraction parameters [169].

An interesting alternative could be the combination of both US and MEF/PEF treatments. Thus, Mello et al. (2019) [170] found that the PEF pre-treatment of orange peel significantly increased the effects on drying rate of ultrasound application during drying. In this sense, the synergistic effect of US and PEF has also been reported in the extraction of betanin from beetroot [171]. PEF/MEF application may induce changes in the structure, e.g., modifying the porosity. This fact can enhance the effectiveness of US application, because the magnitude of the ultrasound effects is greater when the porosity is higher.

#### 5.1.3. Microwaves (MW)

**Figure 1:**
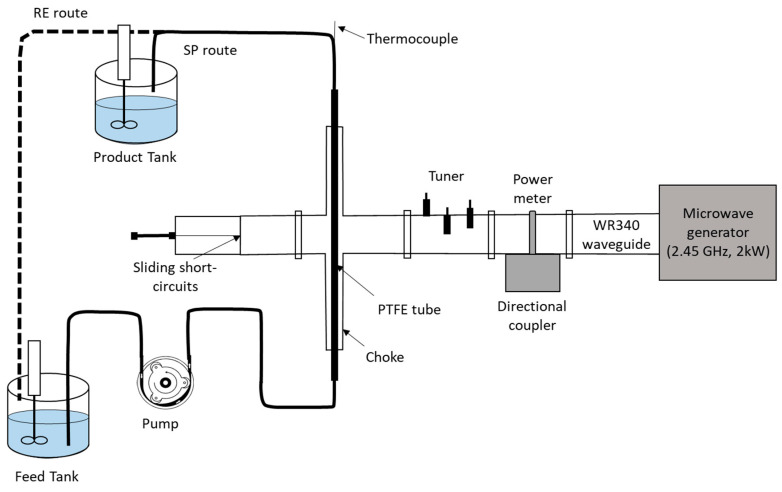
Schema of a continuous-flow MW processing system (adapted from Arrutia et al. 2020) [180].

The microwave-assisted extraction (MAE) process is considered as an emerging technology, particularly for compound extraction from biomaterials by using microwave energy—electromagnetic radiations with a frequency from 0.3 to 300 GHz. Microwaves can penetrate biomaterials and heat them directly by interaction with polar molecules, such as water in the biomaterials [172]. The medium of biomaterials has the ability to absorb and convert microwave energy into thermal energy, dependent on their dielectric properties, which are one of the primary features for its selection as the extracting solvent in the MAE process [103]. Compared with conventional solvent extraction methods, MAE is a novel method providing lower extraction times and solvent consumption [173]. MAE has been widely applied as an effective technique for phenolic compounds extraction from tomatoes [174], grape marc (skins and seeds) [175], pomegranate peels [176] and peanut skins [177], which have high antioxidant activities. Additionally, the MAE process is also considered as a promising method by its effective on receiving higher yield and better quality of extracted fucoidan from brown algae [178], pectin from press residues of berry fruits [94], an acidic polysaccharide from blackberries [179], etc. Arrutia et al. (2020) [180] recently presented a scaled-up continuous flow system for the microwave-assisted extraction of prebiotic hairy pectin from potato waste. Microwave heating is necessary in this process due to its selective and rapid heating, which avoid the deterioration of hairy pectins. Figure 1 shows a schema of this interesting proposal, which represents a concrete step forward towards the implementation of microwave-assisted extraction in the food waste treatment industry. By using the system depicted in Figure 1, it was possible for the authors to recover hairy pectins in the product tank, while starch was concentrated in the feed tank as a sub-product [180].

Microwave has also been applied to food drying to benefit from its quick internal heat generation by the rapid polarization and depolarization of water molecules inside the food material [181], or due to shorter drying times (up to 69%) when compared to hot air drying [182]. In a study by M’hiri et al. (2018) [183], the retention of total phenol and total flavonoids contents was the highest (68.73 and 61.44%, respectively), when combined microwave-air drying (90 W/75 °C) was applied on an industrial lemon by-product. Oil extraction from microwave-dried Hass avocados resulted in high quality and stable avocado oils when compared to those obtained from air-dried avocados [184]. Regarding antioxidant retention, microwave drying was also advantageous for raspberries, for which antioxidant retention was 41.7%, 1.7 times compared to hot air drying alone [185]. The reader is invited to obtain more detailed information about microwave assisted drying in comprehensive reviews found in the literature [186,187,188].

## 6. Processing on Pesticide Residues Reduction

### 6.1. General Food Processing

Processing factors (PF) estimate the effect of processing methods pesticide on residue levels and the disposition of the residues in the processed products, calculated and considered by the Joint FAO/WHO Meeting on Pesticide Residues as follows [189]:

(1) $$\mathrm{PF}=\frac{\mathrm{residues}\;\mathrm{in}\;\mathrm{processed}\;\mathrm{product}\;\left({\mathrm{mg}\;\mathrm{Kg}}^{-1}\right)}{\mathrm{residues}\;\mathrm{in}\;\mathrm{raw}\;\mathrm{agricultural}\;\mathrm{commodity}\;\left({\mathrm{mg}\;\mathrm{Kg}}^{-1}\right)}$$

PF values lower than 1 indicate a reduction in the residue level and higher than 1, a concentration effect [181].

Fruits and vegetables, like other foods, are treated through culinary and food processing before they are consumed. The effects of these handling techniques on pesticide residue levels in fruits and vegetables may be influenced by the physical location of the residues (Section 3.4), as well as the physico-chemical properties, such as solubility, volatility, hydrolytic rate constants, water-octanol partition coefficient and thermal degradation [190]; some shown in Table 1, Table 2, Table 3 and Table 4. Among the main food processing techniques applied to fruit and vegetable products before consumption are accounted washing, peeling, juicing, blanching, fermentation, baking, etc.

Washing with ambient temperature water is the most common procedure in both household and commercial preparations. Washing could be reasonably effective in removing residual pesticides of fruits and vegetables, only if the remaining pesticide concentration is low [191]. The reduction of pesticide residues during washing depends on fruit and vegetable types and their characteristics, as reported by Kar et al. (2012) [192]. For instance, the washing of cabbage and cauliflower with tap water removed about 17–40% of chlorantraniliprole residues [192], while in another study, it eliminated 30–50% of phosalone residues in apple [193]. In the case of tomato, washing with tap water yielded a 10%, 15%, 9%, 19%, 23% and 16% loss of HCB, lindane, p,p-DDT, dimethoate, profenofos, and pirimiphos-methyl, respectively [36]. In addition, the effectiveness of residue reduction by washing depends on the time elapsed since the last pesticide application. For example, a study done by Balinova et al. (2006) [57] demonstrated that a reduction in residues through washing decreased with the sampling time (1 to 3 days). Furthermore, no correlation with the solubility and polarity of compound residues could be found, attributed to the penetration of the residues into the cuticle or tissues of the fruit [57].

Peeling is the most effective process to eliminate residual pesticide before consumption. Balinova et al. (2006) [57] reported that the peeling of peaches for baby food was identified as the most effective treatment for decreasing chlorpyrifos-methyl and fenitrothion residues, two organophosphate pesticides. Moreover, potato peeling allowed 71–75% reduction of organochlorine and organophosphate pesticides residues (malathion, lindane, HCB, p,p-DDT) [34], and chlorpropham, a herbicide and sprout suppressant, 91 to 98% [194]. The effective residue reduction by peeling was also reported by Rawn et al. (2008) [8], since captan residue, a non-systemic organophosphate fungicide, contained on post-harvest apple samples (25.8–5100 ng/g) was removed by about 98% through rinsing and peeling, much more than by rinsing alone (50%). The peeling process also showed its effectiveness in the removal of residues when applied to tomato samples, where the concentration of organochlorines (aldrin, dieldrin, endosulfan, endrin, heptachlor, methoxychlor) and organophosphates (chlorpyriphos, dimethoate, malathion, ethyl-parathion, methyl-parathion) significantly decreased by 28 ± 7% three days after harvest, whereas dimethoate and ethyl-parathion were entirely removed ten days after harvest [195]. In another study, chlorpyriphos contained in red pepper was effectively reduced by more than 93% in peeled samples (from 0.064 to 0.004 mg kg^−1^) [196]. Moreover, in the case of cucumber, the peeling process was the most effective way to reduce carbaryl residues when compared to washing and storage [197].

Juicing is done by pressing fruits or vegetables, sometimes assisted with enzymatic treatment to increase juice yield. During juicing, the combination of supplementary procedures (washing, pressing, sterilization and enzymatic treatment) greatly helps reduce pesticide residues in the matrix. For instance, Li et al. (2015) [198] obtained a 85–95% decrease of β-cypermethrin, chlorpyrifos, tebuconazole, acetamiprid and bendazim in apples matrix during juice processing. In another study, the reduction of HCB, lindane, p,p-DDT, dimethoate, profenofos and pirimiphos-methyl residues ranged from 73 to 78% during tomato juicing [36].

Pesticide residues can also be reduced by other thermal processes such as baking, where undesirable molecules in the tissue co-evaporate with water, or simply degrade [199,200]. For example, detected levels of profenofos residues were reduced from 11.5 ppm to 0.22 ppm in fresh peeled potatoes through microwave-baking, or to 0.19 ppm through oven-baking [201].

Storage of fruits and vegetables prior to processing may represent an important step in degradation of pesticide residues over time, but it varies greatly according to the active ingredient. For instance, Holland et al. (1994) [190] detected the presence of the fungicide dodin and insecticide phosalone in apples after five months of cold storage at 1–3 °C. Athanasopoulos and Pappas (2000) [202] reported differences in the degradation rate of azinphos methyl between apple and lemon, based on their acidity. In general, storage conditions will impact the fate of residues. This is the case of azinphos-ethyl, where its half-life was measured at 10 days for apples on trees, 83 days for apples stored at ambient conditions (18 ± 5 °C, RH~60%), 91 days for apples in controlled-atmosphere rooms, and 136 days for apples in refrigerated rooms (0 ± 0.5 °C, RH~85%) [203].

### 6.2. Changes in Pesticide Residues during Drying

In food processing, drying methods may cause an appreciable decline in pesticide residues as a result of evaporation, degradation and/or co-evaporation. However, different drying methods may impose different effects on pesticides. As no information was found on waste of fruits and vegetables, Table 6 resumes some of the research publications dealing with the impact of drying on pesticide residues in fruits and vegetables. For example, the oven-drying of chili pepper at 60 °C for 35 h caused large reductions (37–49%) of clothianidin, diethofencarb, imidacloprid, and tetraconazole, with processing factors (*PF*) in the range of 0.51–0.63. Conversely, moderate reductions (16 and 22%) of methomyl and methoxyfenozide were observed with *PF* of 0.78 and 0.84, while no reduction of chlorfenapyr, folpet, and indoxacarb was present (*PF* of 0.96–0.98) [204]. Oven-drying also caused a high reduction of both dicarboxymides (iprodione and procymidone) residues (57 and 41%, respectively) in grapes as reported by Cabras et al. (1998) [205]. In another study on sun-dried grapes, residues of chlorpyrifos, diazinon, methidathion and dimethoate decreased by 73, 92, 82 and 39%, respectively [206]. However, drying processes can also lead to a higher concentration of pesticides in their by-products, simply from the loss of water in the treated sample. For example, levels of iprodione residues in sun-dried raisin increased 1.6 times, and that of phosalone 2.8 times compared to fresh fruit [205]. In other studies, the level of triadimenol residues, a metabolite of triadimefon, in sun-dried jujube was found to be more than twice as high as that found in fresh jujube, as a result of degradation of triadimefon into triadimenol during sun-drying [181].

In general, the observed changes during drying are dependent on the type of pesticide compound and may be correlated with the difference in vapor pressure of the mixture. For example, in the article by Noh et al. (2015) [204], the reduction by drying of tetraconazole higher than indoxacarb could be related with its higher vapor pressure (0.18 mPa compared to 2.5 × 10^−5^ mPa, respectively). As well, in Özbey et al. (2017) [206], the 92% decrease of diazinon in dried grapes could be due to its higher vapor pressure compared to the three other pesticides. Moreover, seven pesticide residues in a group of eleven were higher in dried jujube than in the fresh one [181]; this could also be related to their low vapor pressure. Therefore, from the physicochemical properties of pesticides (Table 1, Table 2, Table 3 and Table 4), an estimate of their behavior during drying could be extrapolated (for example, a reduction of chlorpyrifos during drying could be higher than propiconazole, and azoxystrobin).

To end, from the results presented in Table 6, the presence of UV radiation in sun drying seems to enhance the loss of pesticides during drying, such is the case of quinoxyfen in sun-dried grapes compared to oven-dried [214], thiamethoxam and thiacloprid in sun-dried honeysuckle compared to shade natural dried [215], and bifenthrin, which was more affected by sun drying because it is hydrolyzed in the presence of UV rays [218]

### 6.3. Change in Pesticide Residues during Extraction

When extraction methods are applied to foods containing pesticides, the percentage transfer of residues into the solvent will depend on the polarity and solubility of pesticide compounds. Water infusion has been extensively studied regarding to pesticide transfer during tea brewing. For example, the percentage transfer of phosphamidon residue to the tea brew was the highest (33%), followed by dimethoate (26%), monocrotophos (20%), malathion (12%), methyl parathion (10%), quinalphos (8%), and finally chlorpyrifos (3%), as a direct indication of their polarity [225]. Chen et al. (2015) [226] also reported that the transfer rate of nineteen different pesticide residues from tea during brewing was influenced by the octanol−water partition coefficient, and pesticide water solubility. Similarly, Kumar et al. (2005) [227] found that the percentage of propargite residues from manufactured tea to infusion media was in the range of 24–40%, based on the water solubility of residues and their partition coefficient in the solvent.

Other than research on the pesticide transfer during the brewing of tea or other leaves for infusion, there is otherwise little specific investigations on the fate of pesticides during different types of extractions from fruits and vegetables, and none on their waste. Table 7 resumes some of the few publications on the subject. Some of the works listed in Table 7 are just extraction methods developed for analytical determination purposes, but they could indicate the fate of pesticides in similar extraction conditions during the processing of fruit and vegetable wastes. Watanabe et al. (2013) [228] and Iwafune et al. (2014) [229] developed a water-based extraction method for separating pesticides from green pepper, tomato and spinach with high yields. Jaggi et al. (2001) [225] found that most organochlorines, organophosphates, and synthetic pyrethroids residues could be extracted by n-hexane. On the other hand, dimethoate residues were best dissolved in chloroform (96–100%) and those of phosphamidon in dichloromethane (89–95%), two polar solvents. This suggests that the latter residues could possibly be extracted by solvents such as water, whereas less polar pesticides will be best extracted with non-polar solvents, such as n-hexane.

Blanching is a heat treatment for enzyme inactivation, enhancing drying rate and food quality. In addition, it could play an important role in the reduction of polar pesticide residues and toxic constituents in vegetables and fruits. This reduction is explained by the degradation of toxic or pesticide substances that are washed off into the blanching water [230], or by the dissolution of the cuticular waxy layer [231]. Among various processes (washing with tap water, microwave cooking, in-pack sterilization, blanching), hot water blanching was the most effective way to remove deuteratedethylenethiourea, ethylenethiourea, deltamethrin, 3,5-dichloroaniline and boscalid residues in spinach [232].

### 6.4. Impact of Intensification Technologies on Pesticide Reduction

PEF treatment has been applied as an effective method for reducing pesticide residues, their degradation level affected by electric field strength and the number of pulses. Ultrasound may also play an important role by itself or combined to other processes in pesticide residue degradation, such as the degradation rate value of diazinon in apple juice when treated at 500 W, or ultrasound power, which was 1.26 and 1.55 times higher than when treated at 300 W and 100 W, respectively [239]. The ultrasound application time also influenced the pesticide residue degradation and thus, the percentage of degradation of phorate, an organophosphorus pesticide, increased about 16% when the ultrasound treatment time increased from 60 to 120 min at 500 W power [240]. In the case of farm produce (i.e., tomatoes, apples, green peppers, peaches, oranges, and lemons), Al-Taher et al. (2013) [237] found out that sonication used with the washing process would increase pesticide removal from produce surfaces, depending on the washing treatment and on the pesticide. Finally, microwave application could also be considered to be an effective method for the removal of pesticide residues on fruits. Pesticide residues were degraded at higher rates (from 67% to 93%) in jujube fruit dried by microwave drying (700 W, 4 min), when compared to just hot air drying, which was highly corelated with the vapor pressure and water solubility of these pesticide compounds [181].

## 7. Conclusions

The present literature review pointed out that pesticide residues in fruit and vegetable wastes (FVW) processing could pose a problem on human health and environment. The localization of pesticides in foods varies with the nature of molecules, type and portion of plant material and environmental factors, but are usually mostly present in their outer parts of fruits and vegetables. Fruit and vegetable wastes being composed mainly of skin and peel, especially for apple, pepper, tomato, potato, grape and orange wastage, could imply that FVW as raw material for further processing into valuable by-products may be concentrated in agrochemicals.

By-products from FVW are usually bioactive extracts or powders obtained in production lines where air-drying and extraction processes are commonly employed, which may result in increased pesticide/solvent residue concentrations. Drying could concentrate or reduce agrochemicals content depending on the pesticide chemical properties and the type of drying method used. Furthermore, residues in olive oil or apple juice showed great variability upon processing, depending on water solubility of the pesticides and pre-treatments. Thus, the agrochemical content in FVW should be monitored and eventually minimized before or during further by-products processing.

It is surprising how little in-depth research exists on the interaction between pesticide compounds and drying or extraction processes. There are only empirical studies on multi-compound drying of such mixtures and on the application of intensification technologies in by-product processing. In addition, studies on the fate of pesticides during the obtention of extracts from FVW are practically lacking from the literature. This review, being of an exploratory and interpretive nature, thus raised a number of opportunities for future research in the area of the impact of drying and extraction on the fate of pesticide residues in by-products processing from FVW, both in terms of theory development and concept validation.

## Figures and Tables

**Table 1:** Some examples of organochlorines pesticides, which are organic compounds with five or more chlorine atoms [10,11,12,13].

Examples	Physicochemical Characteristics	Health and Environment Risks
Dichlorodiphenyltrichloroethane (DDT)	Melting point: 108.5 °C Solubility in water: 25 × 10^−3^ mg/L (25 °C) Solubility in ethanol (20 × 10^3^ mg/L) Solubility in ether (280 × 10^3^ mg/L) Vapor pressure (20 °C): 2.53 × 10^−5^ Pa	Probable carcinogen Reproductive effect Liver and kidney problem Eye, nose, skin, throat irritant
Captan	Melting point: 178 °C Soluble in water: 3.3 mg/L (25 °C), in acetone: 21 g/L, chloroform: 70 g/L, cyclohexanone: 23 g/L, and in isopropanol: 1.7 g/L. Vapor pressure (20 °C): 13.3 × 10^−5^ Pa.	Probable carcinogen, allergen Induces hyperplasia of the crypt cells Potent eye irritant Mild skin irritant
Lindane	Melting point: 112.5 °C Solubility in water: insoluble. Moderately soluble in ethanol, ether, benzene acetone. Vapor pressure (20 °C): 125.32 × 10^−5^ Pa	Suspected carcinogen Affects central nervous system, and respiratory, reproductive systems.
Endosulfan	Melting point: 70 to 100 °C Solubility in water (22 °C): 0.33 mg/L. Vapor pressure (25 °C): 133.32 × 10^−5^ Pa	Causes DNA damage Potential correlation between endosulfan and leukemia.
Aldrin	Melting point: 104 °C Solubility in water: slightly soluble (0.003%) Vapor Pressure (20 °C): 100 × 10^−4^ Pa	Causes problems with the central nervous system (the brain and spinal cord), and the liver. Eye, skin and mucous membrane irritants.
Dieldrin	Melting point: 176 to 177 °C Solubility in water: 0.186 mg/L at 25–29 °C Vapor Pressure (20 °C): 2.37 × 10^–5^ Pa	Causes problems with the central nervous system (the brain and spinal cord) and the liver. Eye, skin and mucous membrane irritants.
Chlordane	Melting point: 102 to 106 °C Solubility in water: 1 × 10^−3^ mg/L (20 °C) Vapor Pressure (25 °C): 133.32 × 10^−5^ Pa	Chlordane interacts with the human erythrocyte membrane and change its morphology.

**Table 2:** Some examples of organophosphates pesticides, which are esters of phosphoric acid, containing a phosphate group as their basic structural framework [10,13,14,15].

Examples	Physicochemical Characteristics	Health and Environment Risks
Parathion	Melting point: 6 °C Solubility in water: 6.54 mg/L at 24 °C. High solubility in xylene and butanol. Vapor Pressure (20 °C): 503.94 × 10^−5^ Pa	Depressed red blood cell cholinesterase activity, nausea, and headache. Affects central nervous system, blood, respiratory systems, eyes and skin.
Methyl parathion	Melting point: 37 °C.Solubility in water: 37.7 mg/L (20 °C)Vapor Pressure (25 °C): 46.7 × 10^−5^ Pa	Causes neuropsychiatric disorders in humans after chronic exposure as well as hematological and ocular alterations. Reduces cholinesterase levels in the brain, erythrocytes, and plasma.
Malathion	Melting point: 156 to 157 °C Solubility in water: 145 mg/L at 20 °C. Soluble in ethanol and acetone; very soluble in ethyl ether. Vapor Pressure (25 °C): 23.73 × 10^−3^ Pa	Inhibition of Acetylcholinesterase activity Affects central nervous system, respiratory systems. Eye, nose, skin irritant
Diazinon	Flash point: 82 °C. Solubility in water: 40 mg/L Vapor Pressure (20 °C): 111.99 × 10^−4^ Pa	Eye and skin irritant Causes gastrointestinal symptoms.
Glyphosate	Melting point: 184.5 °C Solubility in water: 12 × 10^3^ mg/L (25 °C) Vapor Pressure (25 °C): <1 × 10^−5^ Pa	Probable carcinogen Eye and skin irritant

**Table 3:** Some examples of carbamates pesticides, which are derived from carbamic acid (NH_2_COOH) [10,13,16,17,18].

Examples	Physicochemical Characteristics	Health and Environment Risks
Carbaryl	Melting point: 142 °C Solubility in water: 0.1 mg/L Soluble in most popular organic solvents: dimethylformamide, dimethyl sulfoxide, acetone, cyclohexanone. Vapor Pressure (25 °C): 18.13 × 10^−5^ Pa	Inhibit progesterone biosynthesis of primary human granulose-lutein cells.
Propiconazole	Boiling point: 180 °C Soluble in water: 100 mg/L (25 °C), in hexane: 47 g/L, and in most organic solvents. Completely miscible with ethanol, and acetone. Vapor pressure (25 °C): 13.3 × 10^−5^ Pa.	Skin irritant Liver toxicity and central nervous system effects Adverse changes in erythrocytes
Carbofuran	Melting point: 151 °C Solubility in water: 320 mg/L Highly soluble in N-methyl-2-pyrrolidone, dimethylformamide, dimethyl sulfoxide, acetone, acetonitrile, methylene, chloride, cyclohexanone, benzene, and xylene. Vapor Pressure (33 °C): 2.7 × 10^−3^ Pa	Inhibit acetylcholinesterase. Toxicity affects to vision, growth and predator avoidance skills of fish early life stages.
Propoxur	Melting point: 86 to 92 °C Solubility in water: 1.75 × 10^3^ mg/L (20 °C). Vapor Pressure (20 °C): 39.99 × 10^−5^ Pa	Inhibit of Acetylcholinesterase activity. Probable carcinogen after long-term oral or inhalation exposure.
Aminocarb	Melting point: 93 °C Solubility in water: 0.9 × 10^3^ mg/L. Soluble in polar organic solvents. Moderately soluble in aromatic solvents. Vapor Pressure (20 °C): 2.3 × 10^−3^ Pa	Reduces in immune responsiveness in exposed animals Decrease in activity of acetylcholinesterase. degenerative changes in both liver and kidney

**Table 4:** Some examples of pyrethrins and pyrethroids pesticides, which are synthesized by duplicating the structure of natural pyrethrins, components of pyrethrum flowers are the optically active esters derived from (+)-*trans*-chrysanthemic acid and (+)-*trans*-pyrethroic acid [10,13,19,20].

Examples	Physicochemical Characteristics	Health and Environment Risks
Permethrin	Melting point: 34 °C Solubility in water: 5.5 × 10^−3^ mg/L Vapor Pressure (20 °C): 2.87 × 10^−6^ Pa	Eye, skin, and respiratory irritant. Affects central nervous system.
Cypermethrin	Melting point: 81.3 °C Solubility in water (20 °C): 4 × 10^−3^ mg/L Soluble in ethanol: 337 × 10^3^ mg/L, hexane 103 × 10^3^ mg/L Vapor Pressure (20 °C): 2.27 × 10^−7^ Pa	Causes DNA damage and oxidative stress in gill cells of fish.
Deltamethrin	Melting point: 98 °C Solubility in water: Insoluble Vapor Pressure (25 °C): 2.0 × 10^−6^ Pa	Causes neurotoxicity and liver dysfunction accompanied by elevated reactive oxygen species (ROS) levels.

**Table 5:** Maximum residue limits (MRLs) for fruits and vegetables in Europe, the United States and Canada.

Types of Pesticide	Examples	MRLs (µg kg^−1^)											
		European Commission ^1^				US-FDA ^2^				PCPA Canada ^3^			
		Apple	Potato	Tomato	Strawberry	Apple	Potato	Tomato	Strawberry	Apple	Potato	Tomato	Strawberry
*Organochlorines*	DDT	50	50	50	500	100	---	50	100	For fresh vegetable: 500			
	Captan	10^4^	30	100	100	25 × 10^3^	50	50	2 × 10^4^	5000	---	5000	5000
	Lindane	10	10	10	10	---	500	---	500	Banned			
	Endosulfan	50	50	50	100	---	---	---	---	2000	---	1000	1000
	Aldrin	10	10	10	10	30	100	50	50	---	---	---	---
	Dieldrin	10	10	10	10	30	100	50	50	---	---	---	---
	Chlordane	10	10	10	20	100	100	100	100	---	---	---	---
*Organophosphates*	Parathion	50	50	50	100	---	---	---	---	Banned			
	Methyl parathion	10	10	10	50	---	---	---	---	Banned			
	Malathion	20	20	20	20	8000	8000	8000	8000	2000	500	3000	8000
	Diazinon	10	10	10	50	500	100	750	500	750		750	750
	Glyphosate	100	500	100	2000	200	200	100	200	---	---	---	---
*Carbamates*	Carbaryl	10	10	10	50	12,000	2000	5000	4000	5000	200	5000	7000
	Propiconazole	150	10	300	50	---	---	3000	1300	---	---	3000	1300
	Carbofuran	1	1	2	50	---	---	---	---	---	500		400
	Propoxur	50	50	50	100	---	---	---	---	Banned			
	Aminocarb	---	---	---	---	---	---	---	---	---	---	---	---
*Pyrethrins and pyrethroids*	Permethrin	50	50	50	100	50	50	2000		1000	50	500	---
	Cypermethrin	1000	50	500	100	---	---	---	---	1000	100	300	200
	Deltamethrin	200	300	70	---	200	40	200	---	400	40	300	200

Note: ^1^ EU Pesticides database. Retrieved from http://ec.europa.eu/food/plant/pesticides/eu-pesticides-database/public/?event=homepage&language=EN. ^2^ United States Department of Agriculture. Retrieved from https://www.fas.usda.gov/maximum-residue-limits-mrl-database. ^3^ MRLs for pesticides regulated under the Pest Control Products Act (PCPA). Retrieved from https://www.canada.ca/en/health-canada/services/consumer-product-safety/pesticides-pest-management/public/protecting-your-health-environment/pesticides-food/maximum-residue-limits-pesticides.html.

**Table 6:** Effect of drying methods on residual pesticides in fruits and vegetables.

F&V Produce	Pesticide Compounds	Operation	Conditions	Results	Reference
Apple [193], apple pomace [207]	Phosalone [193], kelthane [207]	Rotating ‘Hatmacker’ drum dryer [193], natural drying [207]	Steam pressure (5 bars), discharge rate (150 L/h), rotation speed (5–76 cm/s) [193]. In the dark, under UV light or sunlight [207].	Phosalone levels were reduced from 22 to 77%. Manufacturers should seek the total elimination of surface residues, i.e., peeling the fruit [193] to improve quality. The loss of kelthane residues was mainly due to volatility rather than photodecomposition [207].	[193,207]
Apricot	Phosalone, iprodione, diazinon, procymidone, bitertanol [208], fenitrothion, dimethoate, omethoate, ziram [209]	Sun drying [208] and ventilated oven [208,209].	Sunlight for 7 days [208] and ventilated oven at 100 °C for 30 min and at 70 °C for 12 h [208,209].	Pesticide residues present in dried fruit were lower than in the fresh fruit (half after sun drying). The exception was phosalone, which increased by 50 (sun-drying) and by 3 times for oven-drying [208]. Omethoate and ziram residues almost doubled after drying, while fenitrothion disappeared and dimethoate remained constant [209].	[208,209]
Chili pepper	Chlorfenapyr, clothianidin, diethofencarb, folpet, imidacloprid, indoxacarb, methomyl, methoxyfenozide and tetraconazole	Oven drying	60 °C for 35 h	Large reductions (37–49%) in clothianidin, diethofencarb, imidacloprid, and tetraconazole. Moderate reductions (16 and 22%) in methomyl and methoxyfenozide, respectively. No effect of drying on chlorfenapyr, folpet, and indoxacarb levels.	[204]
Grape	Iprodione and procymidone [205] Benalaxyl, dimethoate, iprodione, metalaxyl, phosalone, procymidone, vinclozolin [210] Chlorpyrifos, diazinon, methidathion and dimethoate [206] Dimethomorph, famoxadone and cymoxanil [211] Azoxystrobin [212] Pyraclostrobin and metiram [213] Quinoxyfen [214]	Oven drying [205,214] Sun drying & oven drying [206,210] Natural drying [211,213]	70 °C for 24 h [205,214]. No operating conditions [210]. Direct sunlight for 21 days and in an oven at 50 °C for 72 h, at 60 °C for 60 h, at 70 °C for 48 h, at 80°C for 36 h [206]. Shade and outdoors, for 15 days [211] or 25 days [213]. Direct sunlight for 15 days [212]	Iprodione and procymidone decreased by 57 and 41%, respectively [205]. Benalaxyl, phosalone, metalaxyl, and procymidone residues in sun-dried grapes were the same as those on fresh grapes, whereas those of iprodione were higher (1.6 times) and vinclozolin and dimethoate, lower. For the oven-drying process, benalaxyl, metalaxyl, and vinclozolin showed the same residue values in fresh and dried fruits, whereas iprodione and procymidone resides were lower in raisins [210]. Chlorpyrifos, diazinon, methidathion and dimethoate decreased by 73, 92, 82 and 39%, respectively [206]. PF values for raisin processing were 1.03 to 1.14 for dimethomorph, 1.95 to 2.09 for famoxadone, and 1.99 to 1.35 for cymoxanil [211]. Pre-treatment with alkali and sun drying effectively removed a substantial amount of azoxystrobin residues. Commercial production of raisins is, however, carried out with sun drying only [212]. PF values 1.01 to 1.31 for metiram and 1.34 to 1.10 for pyraclostrobin indicated residue concentration after drying [213]. The residue levels in oven dried raisins were comparable to fresh grapes. The lower degradation in the oven-dried sample could be explained by the absence of the degradation effect due to solar radiation [214].	[205,206,210,211,212,213,214]
Honeysuckle (*Lonicera japonica*)	Thiamethoxam and thiacloprid	Three drying methods: sun-, natural (shade)-, and oven drying.	Oven-drying at 30, 40, 50, 60, and 70 °C	59.4–81.0% residue reduction after sun- and oven-drying at 70 °C, higher than for shade- and oven-drying at lower temperatures (at 30 to 60 °C).	[215]
Jujube	Dichlorvos, malathion, chlorpyrifos, triadimefon, hexaconazole, myclobutanil, kresoxim-methyl, tebuconazole, epoxicona-zole, bifenthrin, and cyhalothrin	Microwave drying	Microwave oven (700 W) for 4 min	Degradation rates were from 67% to 93%	[181]
Kumquat candied fruit	Dimethoate, chlorpyrifos, malathion, methidathion and triazophos	Convective drying	60–80 °C	PF of dimethoate, malathion and triazophos after drying were >1, which could be due to the water loss.	[216]
Okra	Malathion, carbaryl [217], endosulfan [217,218], bifenthrin and profenofos [218]	Convective drying [217], sun drying [218]	No specific conditions were found [217,218]	91.8% malathion, 78% carbaryl and 57.4% endosulfan removal [217]. Sun drying helped to decrease endosulfan up to 5.5%, profenos up to 11% and bifenthrin, up to 75%. Bifenthrin was more affected by sun drying because it is hydrolyzed in the presence of UV rays [218].	[217,218]
*Pleurotus ostreatus* mushroom	Carbendazim	Sun drying and freeze-drying	Direct sunlight (sun drying) and at −86 °C with vacuum of 0.06 mbar (freeze-drying)	Direct sun-drying removed higher carbendazim amounts than freeze-drying, with removal rates ranging between 70 and 97%.	[219]
Plum	Bitertanol, diazinon, iprodione, phosalone, procymidone, and vinclozolin [220] Buprofezin, L-cyhalothrin, spirodiclofen, indoxacarb, acetamiprid, imidacloprid, emamectin benzoate [221]	Oven drying [220] Sunlight drying [221]	Temperature: 30 min at 95 °C, 30 min at 90 °C, 16 h at 85 °C [220] Sunlight drying for 26 days with avg. air temp. 17.6 °C, relative hum. 67.3%, solar radiation 546.3 W m^−2^; no rain fell [221].	PF factor was around 3, however pesticide residues were lower or similar in dried than in fresh fruits: phosalone showed the same value, while procymidone, iprodione, and bitertanol were lower (0.6, 2.3 and 3.2 times, respectively). [220]. The insecticide residue reductions during sunlight drying was variable and related to the pesticides’ physico-chemical properties. The whole industrial prune processing has an important reduction effect on pesticide residues [221].	[220,221]
Red pepper	Chlorpyriphos and fenitrothion	Sun/hot air drying	----	Sun or hot air-drying eliminated a 20–30% of residues.	[218,222]
Shiitake mushroom	Carbendazim, thiabendazole, procymidone, bifenthrin, λ-cyhalothrin, and β-cyfluthri	Drying	Sunlight (26–33 °C, 20 days) and hot-air drying (30–53 °C in the first 10 h, 53–60 °C in the last 10 h)	Removal rate of pesticides by sunlight exposure drying (36.2–94.6%) was higher than that of hot-air drying (26.0–68.1%)	[223]
Spring onion	Etofenprox	Drying	Oven (80 °C for 24 h) and freeze-dried (3 days)	Removal rate by oven dried (85.5%) higher than freeze-dried (66.6%)	[224]

**Table 7:** Effect of extraction on residual pesticides in plant-based foods (mainly fruits and vegetables but also including leaves for infusion).

F&V Produce	Pesticide Compounds	Operation	Conditions	Results	Reference
Apples, asparagus, beets, cucumbers, green beans, lettuce, nectarines, peaches, peas, raspberries, spinach, strawberries, tomatoes	Captan, chlorothalonil, iprodione, vinclozolin, endosulfan, permethrin, methoxychlor, malathion, etc.	Water immersion (rinsing)	Ambient temperature for 15–30 s.	A short rinse in tap water reduces pesticide residues on many types of produce. Water solubility of pesticides did not play a significant. The majority of pesticide residue appears to reside on the surface.	[233]
Cabbage, cantaloupe, pear, white potato	Over 33 types of pesticides coming from different families	Accelerated solvent extraction	Dionex ASE 200 extractor; solvent acetone/dichloromethane; 110 °C; 1500 psi; 2 cycles	Accelerated solvent extraction with acetone/dicholoromethane was able to extract a wide range of pesticide residues	[234]
Fruit juice soft drinks (bottles and cans of different brands from 15 European countries)	Over 100 pesticide compounds from 8 different families	Solid-phase extraction (SPE)	HLB cartridges (200 mg) Soft drink samples passed through the cartridges at a flow rate of 3 mL min^−1^.	Carbendazim, thiabendazole, imazalil, prochloraz, malathion, and iprodione were detected in fruit soft drinks, which are mainly those applied to crops as postharvest treatment. The presence of these pesticides in fruit-based soft drinks could be attributed to the use of the peels in the extracts. Therefore, steps should be taken with the aim of removing any traces of pesticides in these products.	[235]
Green pepper, tomato, spinach	Acetamiprid, clothianidin, dinotefuran, flonicamid, imidacloprid, methomyl, pymetrozine, thiacloprid, and thiamethoxam	Water extraction	Please refer to reference.	Water extraction of downsized samples allow quantitative recovery of hydrophilic pesticides	[228]
Green pepper, tomato	CPMF, dinotefuran, CPMA, Nitenpyram, thiamethoxam, clothianidin, imidacloprid, thiaclopridamide, acetamiprid, thiacloprid	Water extraction	Please refer to reference.	This water-based extraction method is convenient to remove pesticides and could be utilized for regular monitoring of neonicotinoid insecticides and their metabolites in high water content crops.	[229]
Peppermint leaves	Malathion, fenitrothion, dimethoate, chlorpyrifos and pirimiphos-ethyl	Infusion	Boiling water (2 g in 100 mL), in 5, 10, 15 and 20 min.	Residues of dimethoate into the infusion was highest (91%), followed by malathion (62%) and fenitrothion (38%)	[236]
Spinach	Boscalid, mancozeb, iprodione, propamocarb, and deltamethrin	Blanching	Sample immersed in hot water (88 °C) for 5 min.	Decreased residue of propamocarb (70%), iprodione, and others by 10 to 58%.	[232]
Tea leaves	Phosphamidon, dimethoate, monocrotophos, malathion, methyl parathion, quinalphos, and chlorpyrifos [185], propargite [187]	Extraction (infusion)	Boiling water (5 g in 200 mL), for 2, 5 and 10 min [185] and for 2 min [187]	Residue to the tea brew: 33%, 26%, 20%, 12%, 10%, 8%, and 3%, respectively [185]; 24–40% in infusion media [187]	[225,227]
Tomato (cherry), and farm produce i.e., tomatoes, apples, green peppers, peaches, oranges, and lemons	Acephate, malathion, carbaryl, bifenthrin, cypermethrin, cyhalothrin, permethrin, chlorothalonil, and imidacloprid	Water immersion (washing)	Pure water (for all produce) or other washing solutions (for Cherry tomatoes) with added chemical compounds (600 rpm for 1 min, at 10 °C forwashing solutions while for pure water, at 5, 10, and 22 °C, w/wo sonication).	Cherry tomatoes washed at 22 °C presented the highest reduction. Residues in contaminated produce decreased from 40 to 90%. Sonication used with the washing process would increase pesticide removal from produce surfaces.	[237]
Thyme and stinging nettle leaves	Fenitrothion, dimethoate, chlorpyrifos and pirimiphos-ethyl	Infusion	Boiling water (2 g in 100 mL), in 5, 10, 15 and 20 min.	The residues of dimethoate (highest water solubility) transferred into the infusions (89–86%), followed by fenitrothion (27–29%), pirimiphos -ethyl (8–14%) and chlorpyrifos (8–8%) during 5 min infusion.	[238]
